# Parameter identification for gompertz and logistic dynamic equations

**DOI:** 10.1371/journal.pone.0230582

**Published:** 2020-04-09

**Authors:** Elvan Akın, Neslihan Nesliye Pelen, Ismail Uğur Tiryaki, Fusun Yalcin

**Affiliations:** 1 Department of Mathematics and Statistics, Missouri University of Science and Technology, Rolla, Missouri, United States of America; 2 Department of Mathematics, Ondokuz Mayıs University, Arts and Science Faculty, Samsun, Turkey; 3 Department of Mathematics, Bolu Abant Izzet Baysal University, Faculty of Arts and Science, Bolu, Turkey; 4 Department of Mathematics, Faculty of Science, Akdeniz University, Antalya, Turkey; IUMPA - Universitat Politecnica de Valencia, SPAIN

## Abstract

In this paper, we generalize and compare Gompertz and Logistic dynamic equations in order to describe the growth patterns of bacteria and tumor. First of all, we introduce two types of Gompertz equations, where the first type 4-paramater and 3-parameter Gompertz curves do not include the logarithm of the number of individuals, and then we derive 4-parameter and 3-parameter Logistic equations. We notice that Logistic curves are better in modeling bacteria whereas the growth pattern of tumor is described better by Gompertz curves. Increasing the number of parameters of Logistic curves give favorable results for bacteria while decreasing the number of parameters of Gompertz curves for tumor improves the curve fitting. Moreover, our results overshadow some of the existing results in the literature.

## Introduction

Most of the growth curves are described by linear, power, parabolic, power-exponential, logistic, log-logistic, von Bertalanffy, Gompertz, and Richards curves; see [1], [2], [3], [4], [5], and [6] for the tumor, [7] for the human fetus, [8] for the human life. A recent research article [8] related with a human life modeled by Gompertz and Mirror Gompertz differential equations are

$$\begin{array}{c} {\left(\text{ln}\;x\right)}^{\prime }=-\beta \;\text{ln}\;x, \end{array}$$ (1)

and

$$\begin{array}{c} x^{\prime }=-\beta \left(1-x\right)\;\text{ln}\;\left(1-x\right), \end{array}$$ (2)

respectively, where *β* is a positive parameter. The well-known logistic equation in the literature, see [9] is given by

$$\begin{array}{c} x^{\prime }=kx(1-\frac{x}{K}), \end{array}$$ (3)

where *k* is the proportionality constant and *K* is the carrying capacity.

In this study, mathematical modeling is applied to the Pseudomonas putida and mammary tumor datas given in [10, 11], respectively. Note that Pseudomonas putida is a bacterium found in most soil and water habitats, and is significant to the environment due to its complex metabolism and ability to control pollution, [12] and [13]. We model their growth patterns by continuous and discrete Gompertz and Logistic curves. To achieve our goal, we derive 4-parameter and 3-parameter Gompertz and Logistic dynamic equations. We first propose two types of Gompertz dynamic equations: The first type Gompertz dynamic equations are motivated by [14]. We contribute two first type continuous Gompertz curves to the literature. All of the discrete Gompertz curves in this type are new. 4-parameter second type Gompertz dynamic equations are motivated by [2] in which only 3-parameter discrete Gompertz curves are considered. 3-parameter second type continuous Gompertz are investigated earlier in [10]. Inspired by [15], we come up with 4-parameter Logistic dynamic equations while 3-parameter Logistic dynamic equations are constructed earlier in [16]. 4-parameter Logistic discrete curves are new. To establish both dynamic equations, we use the variation of constant formulas together with the circle dot multiplication and the circle minus substraction on time scales. We refer readers to [17] and [18] by Bohner and Peterson for the theory of time scales calculus.

The parameters of these models are estimated by NonlinearModelFit function of Wolfram Mathematica 11.0 applying Monte Carlo simulation and our comparison is based on outputs following from the p-values of parameters, adjusted R-squared, and RMSE (root mean square error), RRMSE (Relative Root Mean Square Error), MAPE (Mean Absolute Percent Error), MAE (mean absolute error), U1 (Theil inequality coefficient, Theil’s U1), and U2 (Theil inequality coefficient, Theil’s U2). We use the Mathematica 11 program for the goodness of fit test of the models. Having at least three small values of each determined statistical criterion, the p value less than 0.05 for each parameter, and adjusted R-squared value close to 1 show better performance in terms of goodness of fit.

Outline of this paper is as follows: In Section 2, we introduce the time scales calculus together with some preliminary results. Sections 3 and 4 are related with first and second type Gompertz dynamic equations. In each section we obtain 4-parameter and 3-parameter continuous and discrete Gompertz curves. In Section 6, Logistic dynamic equations are introduced and we explicitly calculate 4-parameter and 3-parameter continuous and discrete Logistic curves. In the last section, we discuss how Gompertz and Logistic curves fit the growth of Pseudomonas putida and mammary tumor and include our conclusion.

## Preliminary results

A *time scale*, T, is an arbitrary nonempty closed subset of the real numbers R. The theory of time scales is to introduce a new calculus so that we can unify the continuous and discrete analysis. Here, we give basic definitions and some essential results without proofs. Nevertheless, we mainly refer readers two books [17] and [18] by Bohner and Peterson and the manuscript [16] by Akin-Bohner and Bohner.

The *forward jump operator* *σ* on T is defined as σ(t)≔inf{s>t:s∈T}∈T, for all t∈T. For this definition we also have σ(∅)=supT. The *backward jump operator* ρonT is defined by ρ(t)≔sup{s<t:s∈T}∈T, for all t∈T. Here, we have ρ(∅)=infT. If *σ*(*t*) > *t*, we say t is *right-scattered*, while if *ρ*(*t*) < *t* we say *t* is *left-scattered*. If *σ*(*t*) = *t*, we say *t* is *right-dense*, while if *ρ*(*t*) = *t* we say *t* is *left-dense*. The *graininess* function μ:T↦[0,∞) is defined by *μ*(*t*): = *σ*(*t*) − *t*. It is apparent that for T=Z, *σ*(*t*) = *t* + 1, *ρ*(*t*) = *t* − 1 and for T=R, *σ*(*t*) = *t*, *ρ*(*t*) = *t*. The set $T^{\kappa }$ is derived from T. If T has left-scattered maximum *m*, then $T^{\kappa }=T-\left\{m\right\}$. Otherwise, $T^{\kappa }=T$. The following notations are also useful: *f*^*σ*^(*t*) = *f*(*σ*(*t*)). Note that $t\in {\left[t_{0},\infty \right)}_{T}=\left[t_{0},\infty \right)\cap T$.

Assume f:T↦R and let $t\in T^{\kappa }$, then we define *f*^Δ^(*t*) to be the number (provided it exists) with the property that given any *ϵ* > 0, there is a neighborhood *U* of *t* such that

$$\begin{array}{c} |\left[f\left(\sigma \left(t\right)\right)-f\left(s\right)\right]-f^{\Delta }\left(t\right)\left[\sigma \left(t\right)-s\right]|\leq \epsilon |\sigma \left(t\right)-s|, \end{array}$$

for all *s* ∈ *U*. *f*^Δ^(*t*) is called the *delta derivative* of *f*(*t*) at *t*. Note that the delta-derivative turns out to be the usual derivative when T=R while it is the forward difference operator when T=Z. If *f* is differentiable at *t*, then *f* is continuous at *t*. If *f* is continuous at *t* and *t* is right- scattered, then *f* is differentiable at *t* with

$$\begin{array}{c} f^{\Delta }\left(t\right)=\frac{f(\sigma (t))-f(t)}{\mu (t)}. \end{array}$$

If *f* is differentiable and t is right-dense, then

$$\begin{array}{c} f^{\Delta }\left(t\right)=\lim_{s\to t}\frac{f(t)-f(s)}{t-s}. \end{array}$$

If *f* is differentiable at *t*, then

$$\begin{array}{c} f^{\sigma }\left(t\right)=f\left(t\right)+\mu \left(t\right)f^{\Delta }\left(t\right). \end{array}$$ (4)

If f,g:T↦R are differentiable at $t\in T^{\kappa }$, then the product fg:T↦R is also differentiable at *t* with

$$\begin{array}{c} {\left(fg\right)}^{\Delta }\left(t\right)=f^{\Delta }\left(t\right)g\left(t\right)+f\left(\sigma \left(t\right)\right)g^{\Delta }\left(t\right). \end{array}$$

If *f* is continuous at each right-dense point t∈T and whenever t∈T is left-dense ${\text{lim}}_{s\to t^{-}}f(s)$ exists as a finite number, then we say that f:T↦ℝ is *rd-continuous*. A function $F:T^{\kappa }\mapsto \mathbb{R}$ is called an *antiderivative* of f:T↦R provided *F*^Δ^(*t*) = *f*(*t*) holds for all $t\in T^{\kappa }$. In this case, we define the integral of *f* by

$$\begin{array}{c} \int_{a}^{t}f\left(s\right)\Delta s=F\left(t\right)-F\left(a\right)\;for\;t\in T. \end{array}$$ (5)

If 1 + *μ*(*t*)*p*(*t*) ≠ 0 for all $t\in T^{\kappa }$, $p:T^{\kappa }\mapsto R$ is called *regressive*. The set of all regressive and rd-continuous functions is denoted by *R*. If 1 + *μ*(*t*)*p*(*t*) > 0 for all $t\in T^{\kappa }$, $p:T^{\kappa }\mapsto R$ is called *positively regressive*. The set of all positively regressive and rd-continuous functions is denoted by *R*^+^.

If *p*, *q* ∈ *R* and *α* is a constant, then we define

$$\begin{array}{c} ⊖p\left(t\right)=-\frac{p(t)}{1+\mu (t)p(t)},\;\;p\left(t\right)⊖q\left(t\right)=\frac{p(t)-q(t)}{1+\mu (t)q(t)}, \end{array}$$ (6)

and

$$\begin{array}{c} \left(p⊕q)(t)=p(t)+q(t)+\mu (t)p(t)q(t\right) \end{array}$$

for all $t\in T^{\kappa }$. Finding a simple formula of the derivative of any power of a function yields to the introduction of a circle dot multiplication. A circle dot multiplication ⊙ is defined in [16] as

$$\begin{array}{c} \left(\alpha ⊙p\right)\left(t\right)=\alpha p\left(t\right)\int_{0}^{1}{\left(1+h\mu \left(t\right)p\left(t\right)\right)}^{\alpha -1}dh. \end{array}$$

Note that ⊖*p* = −*p*, *p* ⊕ *q* = *p* + *q* and *α* ⊙ *p* = *αp* for the continuous case. If *p* is regressive, then we define the exponential function by

$$\begin{array}{c} e_{p}\left(t,s\right)=\text{exp}\;(\int_{s}^{t}\xi_{\mu }\left(p\left(\tau \right)\right)\Delta \tau )\;\;\;for\;s,t\in T, \end{array}$$ (7)

where $\xi_{h}\left(z\right)=\frac{1}{h}Log\left(1+hz\right),h>0$ is the cylinder transformation such that *ξ*_0_(*z*) = *z*. If $p:T^{\kappa }\mapsto R$ is rd-continuous and regressive, then the *exponential function* *e*_*p*_(*t*, *t*_0_) is the unique solution of the IVP

$$\begin{array}{c} x^{\Delta }=p\left(t\right)x,\;\;x\left(t_{0}\right)=1 \end{array}$$

on T for each fixed $t_{0}\in T^{\kappa }.$ For data analysis we need to calculate exponential functions

$$\begin{array}{c} e_{\beta }\left(t,t_{0}\right)=e^{\beta (t-t_{0})},\;e_{⊖\beta }\left(t,t_{0}\right)=e^{-\beta (t-t_{0})}\;\text{when}\;T=R \end{array}$$ (8)

$$\begin{array}{c} \;e_{\beta }\left(t,t_{0}\right)={\left(1+\beta \right)}^{t-t_{0}},\;e_{⊖\beta }\left(t,t_{0}\right)={\left(1+\beta \right)}^{-(t-t_{0})}\;\text{when}\;T=Z \end{array}$$ (9)

for a regressive constant *β*, see Table 2.4 in [17].

We use the following properties of the exponential function *e*_*p*_(*t*, *s*), t,s∈T.

**Theorem 0.1**. *If p*, *q are regressive and* $t_{0}\in T$, *then*

1. *e*_*p*_(*t*, *t*) ≡ *and e*_0_(*t*, *s*) ≡ 1;
2. *e*_*p*_(*σ*(*t*), *s*) = (1 + *μ*(*t*)*p*(*t*))*e*_*p*_(*t*, *s*);
3. $\frac{1}{e_{p}\left(t,s\right)}=e_{⊖p}\left(t,s\right)=e_{p}\left(s,t\right)$;
4. $\frac{e_{p}\left(t,s\right)}{e_{q}\left(t,s\right)}=e_{p⊖q}\left(t,s\right)$;
5. *e*_*p*_(*t*, *s*)*e*_*q*_(*t*, *s*) = *e*_*p*⊕*q*_(*t*, *s*);
6. *if p* > 0 *for all* t∈T, *then e*_*p*_(*t*, *t*_0_) > 0 *for all* t∈T;
7. *if p* ∈ *R*^+^, *then e*_*p*_(*t*, *t*_0_) > 0 *for all* t∈T.

In addition, two of the useful formulas for a circle dot are

$$\begin{array}{c} e_{\alpha ⊙p}\left(t,t_{0}\right)={\left(e_{p}\left(t,t_{0}\right)\right)}^{\alpha }, \end{array}$$ (10)

and

$$\begin{array}{c} 1+\mu \left(\alpha ⊙p\right)={\left(1+\mu p\right)}^{\alpha }, \end{array}$$ (11)

where *p* is a regressive function and *α* is a constant, see [16].

The followings are the variation of constants formulas, see Theorems 2.74 and 2.77 in [17]. The equation

$$\begin{array}{c} x^{\Delta }=p\left(t\right)x+f\left(t\right) \end{array}$$ (12)

is called *regressive* if *x*^Δ^ = *p*(*t*)*x* is regressive (i.e., *p* is regressive) and f:T→R is rd-continuous.

**Theorem 0.2**. *Suppose* (12) *is regressive*. *Let* $t_{0}\in T$ *and* $x_{0}\in R$. *The unique solution of the IVP*

$$\begin{array}{c} x^{\Delta }=p\left(t\right)x+f\left(t\right),\;x\left(t_{0}\right)=x_{0} \end{array}$$

*is given by*

$$\begin{array}{c} x\left(t\right)=e_{p}\left(t,t_{0}\right)x_{0}+\int_{t_{0}}^{t}e_{p}\left(t,\sigma \left(\tau \right)\right)f\left(\tau \right)\Delta \tau . \end{array}$$

**Theorem 0.3**. *Suppose* (12) *is regressive*. *Let* $t_{0}\in T$ *and* $x_{0}\in R$. *The unique solution of the IVP*

$$\begin{array}{c} x^{\Delta }=-p\left(t\right)x^{\sigma }+f\left(t\right),\;x\left(t_{0}\right)=x_{0} \end{array}$$

*is given by*

$$\begin{array}{c} x\left(t\right)=e_{⊖p}\left(t,t_{0}\right)x_{0}+\int_{t_{0}}^{t}e_{⊖p}\left(t,\tau \right)f\left(\tau \right)\Delta \tau . \end{array}$$

## First type gompertz dynamic equations

In this section, we will introduce Gompertz dynamic curves motivated by the 4-parameter Gompertz curve

$$\begin{array}{c} \omega \left(t\right)=B+A\;\text{exp}\;(-\text{exp}\left(-K\left(t-t_{0}\right)\right),t\in R \end{array}$$ (13)

given in [19] for the growth curve analyses of bacterial counts. Here, *K* can be found as the growth rate coefficient, *t*_0_ is the initial time, *A* + *B* is the carrying capacity of the environment for the population. To explain the carrying capacity notion we can say that every environment has its own limits, therefore it is impossible for species to grow up infinitely. Thus, the number of the population should be finite.

In order to obtain the Gompertz model in the continuous case, we differentiate Eq (13) and obtain

$$\begin{array}{ccc} \omega^{\prime } & = & AK\;\text{exp}\left\{-\text{exp}\left\{-K\left(t-t_{0}\right)\right\}\right\}\;\text{exp}\left\{-K\left(t-t_{0}\right)\right\}\\ \; & = & \left[\omega \left(t\right)-B\right]K\;\text{exp}\left\{-K\left(t-t_{0}\right)\right\}. \end{array}$$

In addition, note that we have

$$\begin{array}{ccc} e_{⊖(K⊙⊖e_{⊖K})}\left(t,t_{0}\right) & = & \frac{1}{e_{K⊙\;⊖e_{⊖K}}\left(t,t_{0}\right)}={\left(\frac{1}{e_{⊖e_{⊖K}}\left(t,t_{0}\right)}\right)}^{K}\\ & = & {\left(e_{e_{⊖K}}\left(t,t_{0}\right)\right)}^{K}=e_{K⊙e_{⊖K}}\left(t,t_{0}\right) \end{array}$$ (14)

on ${\left[t_{0},\infty \right)}_{T}$, where we use Theorem 0.1 and (10). Since *e*_⊖*K*_(*t*, *t*_0_) = *e*^−*K*(*t*−*t*_0_)^ for t∈R, and (14) holds, then we obtain

$$\begin{array}{ccc} e_{⊖(K⊙⊖e_{⊖K})}\left(t,t_{0}\right) & = & \text{exp}{\left\{\frac{\text{exp}\;(-K\left(t-t_{0}\right))}{K}-\frac{1}{K}\right\}}^{-K}\\ & = & e\;\text{exp}\{-\text{exp}\{-K\left(t-t_{0}\right)\}\},t\in R. \end{array}$$ (15)

Motivated by the calculation above, we have the following initial value problem modeling 4-parameter Gompertz curve on time scales.

**Theorem 0.4**. *The initial value problem*

$$\begin{array}{c} \begin{array}{ccc} \omega^{\Delta }=-(K⊙⊖e_{⊖K}\left(t,t_{0}\right))\omega^{\sigma }+B\left(K⊙⊖e_{⊖K}\left(t,t_{0}\right)\right) & & \\ \omega \left(t_{0}\right)=\omega_{0} & \end{array} \end{array}$$ (16)

*has the solution of the form*

$$\begin{array}{c} \omega =B+\left(\omega_{0}-B\right)e_{K⊙e_{⊖K}}\left(t,t_{0}\right) \end{array}$$

$t\in {\left[t_{0},\infty \right)}_{T}$, *where K is the growth rate and t*_0_ *is the initial time*, *ω*_0_ *is the value of the function at the initial time and B is the coefficient that has an impact on carrying capacity*.

*Proof*. We notice that the positivity of *K* implies the positivity of $e_{⊖K}$ by Theorem 0.1. Since $1+\mu \left(⊖e_{⊖K}\right)=\frac{1}{1+\mu e_{⊖K}}>0$, we have the positively regressivity of ⊖*e*_⊖*K*_. Since 1 + *μ*(*K*⊙⊖*e*_⊖*K*_) = (1 + *μ*(⊖*e*_⊖*K*_))^*K*^ > 0 by (11), the dynamic equation in the IVP (16) is regressive. Therefore, we apply Theorem 0.3 and obtain the unique solution for $t\in {\left[t_{0},\infty \right)}_{T}$

$$\begin{array}{ccc} \omega & = & e_{⊖(K⊙⊖e_{⊖K})}\left(t,t_{0}\right)\omega_{0}+B\int_{t_{0}}^{t}e_{⊖(K⊙⊖e_{⊖K})}\left(t,\tau \right)\left(K⊙⊖e_{⊖K}\left(\tau ,t_{0}\right)\right)\Delta \tau \\ & = & e_{K⊙e_{⊖K}}\left(t,t_{0}\right)\omega_{0}\\ & & +Be_{K⊙⊖e_{⊖K}}\left(t_{0},t\right)\int_{t_{0}}^{t}e_{K⊙⊖e_{⊖K}}\left(\tau ,t_{0}\right)\left(K⊙⊖e_{⊖K}\left(\tau ,t_{0}\right)\right)\Delta \tau \\ & = & e_{K⊙e_{⊖K}}\left(t,t_{0}\right)\omega_{0}+Be_{K⊙⊖e_{⊖K}}\left(t_{0},t\right)\int_{t_{0}}^{t}{\left(e_{K⊙⊖e_{⊖K}}\left(\tau ,t_{0}\right)\right)}^{\Delta }\Delta \tau \\ & = & e_{K⊙e_{⊖K}}\left(t,t_{0}\right)\omega_{0}+Be_{K⊙⊖e_{⊖K}}\left(t,t_{0}\right)[e_{K⊙⊖e_{⊖K}}\left(t,t_{0}\right)-1]\\ & = & B+\left(\omega_{0}-B\right)e_{K⊙e_{⊖K}}\left(t,t_{0}\right), \end{array}$$ (17)

where we use (14) and Theorem 0.1.

**Example 0.5**. Let T=R. Then the continuous Gompertz curve

$$\begin{array}{c} \begin{array}{cc} \omega =B+e\left(\omega_{0}-B\right)\;\text{exp}\left\{-\text{exp}\left\{-K\left(t-t_{0}\right)\right\}\right\} & \end{array} \end{array}$$ (18)

is obtained from (17) for $t\in {\left[t_{0},\infty \right)}_{R}$ by using Eqs (8) and (15). This is compatible with the continuous Gompertz growth curve (13) by taking *A* = *e*(*ω*_0_ − *B*) in (13).

**Example 0.6**. Let T=Z. Since *e*_⊖*K*_(*t*, *t*_0_) = (1 + *K*)^−(*t*−*t*_0_)^ for $t\in {\left[t_{0},\infty \right)}_{Z}$ by (9), (14) yields

$$\begin{array}{c} \begin{array}{ccc} e_{K⊙e_{⊖K}}\left(t,t_{0}\right) & = & {\left[e_{e_{⊖K}}\left(t,t_{0}\right)\right]}^{K}\\ & = & {\left[\text{exp}\;(\sum_{s=t_{0}}^{t-1}\text{ln}\;(1+\frac{1}{{\left(1+K\right)}^{s-t_{0}}}))\right]}^{K}\\ & = & {\left[\text{exp}\;(\text{ln}\;(\prod_{s=t_{0}}^{t-1}1+\frac{1}{{\left(1+K\right)}^{s-t_{0}}}))\right]}^{K}\\ & = & {\left[\prod_{s=t_{0}}^{t-1}\frac{1+{\left(1+K\right)}^{s-t_{0}}}{{\left(1+K\right)}^{s-t_{0}}}\right]}^{K},\;t\in {\left[t_{0},\infty \right)}_{Z} \end{array} \end{array}$$

and so

$$\begin{array}{c} e_{⊖(K⊙⊖e_{⊖K})}\left(t,t_{0}\right)={\left[\prod_{\tau =t_{0}}^{t-1}\frac{{\left(1+K\right)}^{\tau -t_{0}}}{1+{\left(1+K\right)}^{\tau -t_{0}}}\right]}^{-K} \end{array}$$ (19)

for $t\in {\left[t_{0},\infty \right)}_{Z}$. Thus, the discrete Gompertz growth curve

$$\begin{array}{c} \omega =B+\left(\omega_{0}-B\right){\left[\prod_{\tau =t_{0}}^{t-1}\frac{1+{\left(1+K\right)}^{\tau -t_{0}}}{{\left(1+K\right)}^{\tau -t_{0}}}\right]}^{K} \end{array}$$ (20)

again follows from (17) for $t\in {\left[t_{0},\infty \right)}_{Z}$.

Motivated by the first variation of constant formula, Theorem 0.2, we derive another Gompertz curve on time scales.

**Theorem 0.7**. *The initial value problem*

$$\begin{array}{c} \begin{array}{ccc} \omega^{\Delta }=⊖\left(K⊙e_{⊖K}\left(t,t_{0}\right)\right)\omega +B\left(⊖\left(K⊙e_{⊖K}\left(t,t_{0}\right)\right)\right) & & \\ \omega \left(t_{0}\right)=\omega_{0} & \end{array} \end{array}$$ (21)

*has the solution of the form*

$$\begin{array}{c} \omega =\left(\omega_{0}+B\right)e_{⊖(K⊙e_{⊖K})}\left(t,t_{0}\right)-B \end{array}$$ (22)

*for* $t\in {\left[t_{0},\infty \right)}_{T}$, *where K is the decay rate coefficient and regeressive*, *t*_0_ *is the initial time*, *ω*_0_ *is the value of the function at the initial time and B is the coefficient that has an impact on carrying capacity*.

*Proof*. Since *K* is regressive, *e*_⊖*K*_ is also regressive by (7). The dynamic equation in the IVP (21) is regressive. Then, in order to obtain the unique solution (22) we apply Theorem 0.3 for $t\in {\left[t_{0},\infty \right)}_{T}$

$$\begin{array}{ccc} \omega & = & e_{⊖(K⊙e_{⊖K})}\left(t,t_{0}\right)\omega_{0}+B\int_{t_{0}}^{t}e_{⊖(K⊙e_{⊖K})}\left(t,\sigma \left(\tau \right)\right)\left(⊖\left(K⊙e_{⊖K}\left(\tau ,t_{0}\right)\right)\right)\Delta \tau \\ & = & e_{⊖(K⊙e_{⊖K})}\left(t,t_{0}\right)\omega_{0}\\ & & -Be_{⊖(K⊙e_{⊖K})}\left(t,t_{0}\right)\int_{t_{0}}^{t}e_{K⊙e_{⊖K}}\left(\tau ,t_{0}\right)\left(K⊙e_{⊖K}\left(\tau ,t_{0}\right)\right)\Delta \tau \\ & = & e_{⊖(K⊙e_{⊖K})}\left(t,t_{0}\right)\omega_{0}-Be_{⊖(K⊙e_{⊖K})}\left(t,t_{0}\right)\int_{t_{0}}^{t}{\left(e_{K⊙e_{⊖K}}\left(\tau ,t_{0}\right)\right)}^{\Delta }\Delta \tau \\ & = & e_{⊖(K⊙e_{⊖K})}\left(t,t_{0}\right)\omega_{0}-Be_{⊖(K⊙e_{⊖K})}\left(t,t_{0}\right)[e_{K⊙e_{⊖K}}\left(t,t_{0}\right)-1]\\ & = & \left(\omega_{0}+B\right)e_{⊖(K⊙e_{⊖K})}\left(t,t_{0}\right)-B, \end{array}$$

where we use (14) and Theorem 0.1.

**Example 0.8**. Let T=R. Then the alternative continuous Gompertz curve

$$\begin{array}{c} \begin{array}{cc} \omega =\frac{1}{e}\left(\omega_{0}+B\right)\;\text{exp}\left\{\text{exp}\left\{-K\left(t-t_{0}\right)\right\}\right\}-B & \end{array} \end{array}$$ (23)

is obtained from (22) for t∈R. It is worth to mention that Eq (23) is a new Gompertz curve in the continuous case.

**Example 0.9**. Let T=Z. Then using (19), we have

$$\begin{array}{c} e_{⊖(K⊙e_{⊖K})}\left(t,t_{0}\right)={\left[\prod_{\tau =t_{0}}^{t-1}\frac{{\left(1+K\right)}^{\tau -t_{0}}}{1+{\left(1+K\right)}^{\tau -t_{0}}}\right]}^{K} \end{array}$$

for $t\in {\left[t_{0},\infty \right)}_{Z}$. Since

$$\begin{array}{c} e_{⊖(K⊙e_{⊖K})}=\frac{1}{e_{K⊙e_{⊙K}}}={\left[\frac{1}{e_{e_{⊖K}}}\right]}^{K}, \end{array}$$

the alternative discrete Gompertz growth curve

$$\begin{array}{c} \omega =\left(\omega_{0}+B\right){\left[\prod_{\tau =t_{0}}^{t-1}\frac{{\left(1+K\right)}^{\tau -t_{0}}}{1+{\left(1+K\right)}^{\tau -t_{0}}}\right]}^{K}-B \end{array}$$ (24)

again follows from (22) for $t\in {\left[t_{0},\infty \right)}_{Z}$.

The Gompertz growth curve (23) is given as 4-parameter Gompertz growth curve in [19]. From this point of view, the 3-parameter Gompertz growth curve on time scales

$$\begin{array}{c} \omega =\omega_{0}e_{K⊙e_{⊖K}}\left(t,t_{0}\right) \end{array}$$ (25)

is obtained from (17) when *B* = 0, and so the 3-parameter continuous and discrete Gompertz curves are

$$\begin{array}{c} \omega =e\omega_{0}\text{exp}\left\{-\text{exp}\left\{-K\left(t-t_{0}\right)\right\}\right\} \end{array}$$ (26)

for $t\in {\left[t_{0},\infty \right)}_{R}$ and for $t\in {\left[t_{0},\infty \right)}_{Z}$

$$\begin{array}{c} \omega =\omega_{0}{\left[\prod_{\tau =t_{0}}^{t-1}\frac{1+{\left(1+K\right)}^{\tau -t_{0}}}{{\left(1+K\right)}^{\tau -t_{0}}}\right]}^{K}, \end{array}$$ (27)

respectively. From (22) when *B* = 0, the alternative 3-parameter continuous and discrete Gompertz curves

$$\begin{array}{c} \omega =\frac{1}{e}\omega_{0}\text{exp}\left\{\text{exp}\left\{-K\left(t-t_{0}\right)\right\}\right\} \end{array}$$ (28)

and

$$\begin{array}{c} \omega =\omega_{0}{\left[\prod_{\tau =t_{0}}^{t-1}\frac{1+{\left(1+K\right)}^{\tau -t_{0}}}{{\left(1+K\right)}^{\tau -t_{0}}}\right]}^{-K}, \end{array}$$ (29)

are gained to the literature, respectively.

### The zwietering modification of gompertz growth curve

The Gompertz growth curve is reparameterized in order to model the bacteria growth population in food and is stated as

$$\begin{array}{c} w=A\;\text{exp}\{-\text{exp}\left\{\frac{eK_{z}}{A}\left(T-t\right)+1\right\}\} \end{array}$$ (30)

in [14] for t∈R, where *K*_*z*_ is the absolute growth rate at time *T*, so called lag time, which is interpreted as the time between when a microbial population is transferred to a new habitat recovers and when a considerable cell division occurs.

In order to find a corresponding dynamic model, we rewrite (30) as

$$\begin{array}{c} w=A{\left(\text{exp}\left\{-\text{exp}\left\{-\frac{eK_{z}}{A}t\right\}\right\}\right)}^{e^{\frac{eK_{z}}{A}T+1}} \end{array}$$ (31)

so that we can use the property of circle dot (10) for the unification the continuous and discrete cases. Therefore, using (15) and (14) yield the dynamic Zwietering Modification Gompertz curve

$$\begin{array}{ccc} w & = & Ae^{-(\frac{eK_{z}}{A}T+1)}{\left(e_{⊖(\frac{eK_{z}}{A}⊙⊖e_{⊖\frac{eK_{z}}{A}})}\left(t,0\right)\right)}^{e^{\frac{eK_{z}}{A}T+1}}\\ & = & Ae^{-(\frac{eK_{z}}{A}T+1)}e_{e^{\left(\frac{eK_{z}}{A}T+1\right)}⊙⊖\left(\frac{eK_{z}}{A}⊙⊖e_{⊖\frac{eK_{z}}{A}}\right)}\left(t,0\right)\\ & = & Ae^{-(\frac{eK_{z}}{A}T+1)}e_{e^{\left(\frac{eK_{z}}{A}T+1\right)}⊙\left(\frac{eK_{z}}{A}⊙e_{⊖\frac{eK_{z}}{A}}\right)}\left(t,0\right). \end{array}$$ (32)

Therefore, we claim that (32) is the solution of the IVP

$$\begin{array}{c} \begin{array}{ccc} \omega^{\Delta }=\left(e^{\left(\frac{eK_{z}}{A}T+1\right)}⊙\left(\frac{eK_{z}}{A}⊙e_{⊖\frac{eK_{z}}{A}}\right)\right)w & & \\ \omega \left(0\right)=Ae^{-(\frac{eK_{z}}{A}T+1)}. & \end{array} \end{array}$$ (33)

Since (30) is the continuous modified Gompertz growth curve, the discrete modified Gompertz growth curve follows from (32).

**Example 0.10**. Let T=Z. Then the discrete Zwietering modification of Gompertz growth curve is

$$\begin{array}{c} \omega =Ae^{-(\frac{eK_{z}}{A}T+1)}{\left[\prod_{\tau =0}^{t-1}\frac{{\left(1+\frac{eK_{z}}{A}\right)}^{\tau }}{1+{\left(1+\frac{eK_{z}}{A}\right)}^{\tau }}\right]}^{-\frac{eK_{z}}{A}e^{\left(\frac{eK_{z}}{A}T+1\right)}} \end{array}$$ (34)

obtained from (19) for $t\in {\left[0,\infty \right)}_{Z}$.

### Gompertz-laird growth curve

This model is mainly used for the modeling of tumor growth. The Laird re-parameterization prevails even today as the most frequently fitted Gompertz version in cancer research, and is now also commonly fitted to growth data in other fields such as those of domestic (e.g. poultry and livestock, marine (e.g. molluscs, fish, and dolphins) animals.

The continuous Gompertz-Laird growth curve is given by

$$\begin{array}{c} \begin{array}{cc} \omega =\omega_{0}e^{-\frac{L}{K}\left(e^{-Kt}-1\right)} & \end{array} \end{array}$$

for $t\in {\left[0,\infty \right)}_{R}$ in [14], which is equivalent to

$$\begin{array}{c} w=w_{0}e^{\frac{L}{K}}{\left(e^{-e^{-Kt}}\right)}^{\frac{L}{K}} \end{array}$$ (35)

for $t\in {\left[0,\infty \right)}_{R}$, where the parameter *L* describes the initial specific growth rate that is not a notion that measures the relative growth or absolute growth. More precisely, we can say that the absolute growth rate at *t* = 0 is *ω*_0_.*L* Thus, the term *L* can be described as division of the initial absolute growth rate with the initial value.

Similarly, by using (15) we obtain the Gompertz-Laird growth curve on time scales as

$$\begin{array}{ccc} w & = & w_{0}{\left(e_{⊖(K⊙⊖e_{⊖K})}\left(t,0\right)\right)}^{\frac{L}{K}}\\ & = & w_{0}e_{\frac{L}{K}⊙\left(⊖\left(K⊙⊖e_{⊖K}\right)\right)}\left(t,0\right)\\ & = & w_{0}e_{\frac{L}{K}⊙\left(K⊙e_{⊖K}\right)}\left(t,0\right) \end{array}$$ (36)

for $t\in {\left[0,\infty \right)}_{T}$ and so (36) is the solution of the IVP

$$\begin{array}{c} \begin{array}{ccc} w^{\Delta }=\frac{L}{K}⊙\left(K⊙e_{⊖K}\right)w & & \\ \omega \left(0\right)=\omega_{0}. & \end{array} \end{array}$$ (37)

Since (35) is the continuous Gompertz-Laird growth curve, the following example gives the discrete Gompertz-Laird growth curve.

**Example 0.11**. Let T=Z. Then we obtain

$$\begin{array}{c} \omega =\omega_{0}{\left[\prod_{\tau =0}^{t-1}\frac{{\left(1+K\right)}^{\tau }}{1+{\left(1+K\right)}^{\tau }}\right]}^{-L} \end{array}$$ (38)

as the discrete Gompertz-Laird growth curve for $t\in {\left[t_{0},\infty \right)}_{Z}$, where we use again (19).

If *L* = *Km* in (36), the **Zweifel and Lasker** re-parametrization dynamic equation is obtained for studying fish growth. Moreover, the continuous and discrete curves are given in (35), (38), respectively. Similarly, if $L=\text{ln}\;{\left(\frac{A}{\omega_{0}}\right)}^{K}$ in (36), we derive the dynamic form of **Simpler *W*_0_ form** of Gompertz Laird growth curve which prevails on. Moreover, the continuous and discrete curves are given in (35) and (38), respectively. Note that all of first type Gompertz curves in the discrete case are new.

## Second type gompertz dynamic equations

It is clear that (13) is not a Gompertz model when the dependent variable is log-transformed. In this subsection, we will derive Gompertz dynamic equations involving logarithmic functions. This idea of the derivation of Gompertz dynamic equations is inspired from the Gompertz difference equation

$$\begin{array}{c} \text{ln}\;G(t+1)=a+b\;\text{ln}\;G(t),\;t\in Z \end{array}$$ (39)

where a is taken as the growth rate and b is taken as the exponential rate of growth deceleration, which was firstly given by Bassukas et. al. [3]. The equivalent form of (39) is given by

$$\begin{array}{c} \Delta \text{ln}\;G(t)=a+(b-1)\text{ln}\;G(t),\;t\in Z \end{array}$$ (40)

and so the continuous version becomes

$$\begin{array}{c} {\left[\text{ln}\;G\left(t\right)\right]}^{{}^{\prime }}=a+\left(b-1\right)\;\text{ln}\;G\left(t\right),\;t\in R. \end{array}$$ (41)

Unifying (40) and (41), we end up by

$$\begin{array}{c} {\left[\text{ln}\;G\left(t\right)\right]}^{\Delta }=a+\left(b-1\right)\;\text{ln}\;G\left(t\right),\;t\in T. \end{array}$$ (42)

By the second variation of constants formula, Theorem 0.3, we get the alternative dynamic equation

$$\begin{array}{c} {\left[\text{ln}\;G\left(t\right)\right]}^{\Delta }=a-\left(b-1\right)\;\text{ln}\;G\left(\sigma \left(t\right)\right),\;t\in T. \end{array}$$ (43)

Notice that Eq (42) turns out to be (39) when T=Z while (43) turns out to be

$$\begin{array}{c} \text{ln}\;G\left(t+1\right)=\frac{a}{b}+\frac{\text{ln}\;G(t)}{b}, \end{array}$$ (44)

for a nonzero constant *b* and when T=R, we obtain the following Gompertz differential equation from the Gompertz dynamic Eq (43) as

$$\begin{array}{c} {\left[\text{ln}\;G\left(t\right)\right]}^{{}^{\prime }}=a-\left(b-1\right)\;\text{ln}\;G\left(t\right), \end{array}$$ (45)

which is equivalent to Gompertz differential Eq (1) when *a* = 0, *b* − 1 = *β*, and *G* = *x* in (45). In [8], Gompertz differential Eq (41) with *a* = 0 is called the Mirror Gompertz differential equation, and is equivalent to (2) when *a* = 0, *b* − 1 = *β*, and *G* = 1 − *x* in (41).

From now on, we take *a* = *α* and *b* − 1 = *β* in (42) and (43) and assume

$$\begin{array}{c} \text{ln}\;G\left(t_{0}\right)=g_{0}, \end{array}$$ (46)

where *g*_0_ is a real number. The following theorems yield the second type Gompertz dynamic curves.

**Theorem 0.12**. *Suppose that β is regressive and α > 0*. *Then the solution of the IVP* (42)–(46) *is given by*

$$\begin{array}{c} G\left(t\right)=\text{exp}\;(e_{\beta }\left(t,t_{0}\right)[g_{0}+\alpha \int_{t_{0}}^{t}e_{\beta }\left(t_{0},\sigma \left(\tau \right)\right)\Delta \tau ]) \end{array}$$ (47)

*for* $t\in {\left[t_{0},\infty \right)}_{T}$.

*Proof*. If ln *G*(*t*) is taken as *u*(*t*), then the IVP (42)–(46) becomes *u*^Δ^(*t*) = *α* + *βu*(*t*), *u*(*t*_0_) = *g*_0_, $t\in {\left[t_{0},\infty \right)}_{T}$. Then by Theorems 0.1 and 0.2, we obtain

$$\begin{array}{c} \begin{array}{ccc} u(t) & = & e_{\beta }\left(t,t_{0}\right)g_{0}+\alpha \int_{t_{0}}^{t}e_{\beta }\left(t,\sigma \left(\tau \right)\right)\Delta \tau \\ & = & e_{\beta }\left(t,t_{0}\right)[g_{0}+\alpha \int_{t_{0}}^{t}e_{⊖\beta }\left(t,t_{0}\right)e_{\beta }\left(t,\sigma \left(\tau \right)\right)\Delta \tau ]\\ & = & e_{\beta }\left(t,t_{0}\right)[g_{0}+\alpha \int_{t_{0}}^{t}e_{\beta }\left(t_{0},\sigma \left(\tau \right)\right)\Delta \tau ],\;t\in {\left[t_{0},\infty \right)}_{T}. \end{array} \end{array}$$

Since *G* = *e*^*u*^, (47) is obtained as the solution of the IVP (42)–(46).

**Theorem 0.13**. *Suppose β is regressive and α* > 0. *Then the solution of the IVP* (43)–(46) *is given by*

$$\begin{array}{c} G\left(t\right)=\text{exp}\;(e_{⊖\beta }\left(t,t_{0}\right)[g_{0}+\alpha \int_{t_{0}}^{t}e_{⊖\beta }\left(t_{0},\tau \right)\Delta \tau ]) \end{array}$$ (48)

*for* $t\in {\left[t_{0},\infty \right)}_{T}$.

*Proof*. If ln *G*(*t*) is taken as *u*(*t*), then the IVP (43)–(46) turns out to be *u*^Δ^(*t*) = *α*−*βu*^*σ*^(*t*), *u*(*t*_0_) = *g*_0_, $t\in {\left[t_{0},\infty \right)}_{T}$. Again, Theorems 0.1 and 0.3 yield

$$\begin{array}{c} \begin{array}{ccc} u(t) & = & e_{⊖\beta }\left(t,t_{0}\right)g_{0}+\alpha \int_{t_{0}}^{t}e_{⊖\beta }\left(t,\tau \right)\Delta \tau \\ & = & e_{⊖\beta }\left(t,t_{0}\right)[g_{0}+\alpha \int_{t_{0}}^{t}e_{\beta }\left(t,t_{0}\right)e_{⊖\beta }\left(t,\tau \right)\Delta \tau ]\\ & = & e_{⊖\beta }\left(t,t_{0}\right)[g_{0}+\alpha \int_{t_{0}}^{t}e_{⊖\beta }\left(t_{0},\tau \right)\Delta \tau ] \end{array} \end{array}$$

for $t\in {\left[t_{0},\infty \right)}_{T}$. Since *G* = *u*, (48) is obtained as the solution of the IVP (43)–(46).

**Example 0.14**. Let T=R. Then the solutions of the IVPs (42)–(46) and (43)–(46) are

$$\begin{array}{c} \begin{array}{ccc} G(t) & = & \text{exp}\;(e^{\beta (t-t_{0})}[g_{0}+\alpha \int_{t_{0}}^{t}e^{-\beta (\tau -t_{0})}d\tau ])\\ & = & \text{exp}\;(e^{\beta (t-t_{0})}[g_{0}+\frac{\alpha }{\beta }\left(1-e^{-\beta (t-t_{0})}\right)])\\ & = & \text{exp}\;((g_{0}+\frac{\alpha }{\beta })e^{\beta (t-t_{0})}-\frac{\alpha }{\beta }), \end{array} \end{array}$$ (49)

and

$$\begin{array}{c} \begin{array}{ccc} G(t) & = & \text{exp}\;(e^{-\beta (t-t_{0})}[g_{0}+\alpha \int_{t_{0}}^{t}e^{\beta (\tau -t_{0})}d\tau ])\\ & = & \text{exp}\;(e^{-\beta (t-t_{0})}[g_{0}+\frac{\alpha }{\beta }\left(e^{\beta (t-t_{0})}-1\right)])\\ & = & \text{exp}\;((g_{0}-\frac{\alpha }{\beta })e^{-\beta (t-t_{0})}+\frac{\alpha }{\beta }), \end{array} \end{array}$$ (50)

respectively for *t* ∈ [*t*_0_, ∞) and here we use (8).

**Example 0.15**. Let T=Z. Then the solutions of the IVPs (42)–(46) and (43)–(46) are

$$\begin{array}{c} \begin{array}{ccc} G(t) & = & \text{exp}\;(e_{\beta }\left(t,t_{0}\right)[g_{0}+\alpha \int_{t_{0}}^{t}e_{\beta }\left(t_{0},\sigma \left(\tau \right)\right)\Delta \tau ])\\ & = & \text{exp}\;({\left(\beta +1\right)}^{\left(t-t_{0}\right)}[g_{0}+\alpha \sum_{\tau =t_{0}}^{t-1}{\left(1+\beta \right)}^{-(\tau +1-t_{0})}])\\ & = & \text{exp}\;({\left(\beta +1\right)}^{\left(t-t_{0}\right)}[g_{0}+\alpha {\left(1+\beta \right)}^{-(t-t_{0})}\sum_{\tau =t_{0}}^{t-1}{\left(1+\beta \right)}^{\tau -t_{0}}])\\ & = & \text{exp}\;({\left(\beta +1\right)}^{\left(t-t_{0}\right)}[g_{0}+\alpha {\left(1+\beta \right)}^{-(t-t_{0})}\frac{{\left(1+\beta \right)}^{t-t_{0}}-1}{\beta }])\\ & = & \text{exp}\;((g_{0}+\frac{\alpha }{\beta }){\left(\beta +1\right)}^{t-t_{0}}-\frac{\alpha }{\beta }) \end{array} \end{array}$$ (51)

and

$$\begin{array}{c} \begin{array}{ccc} G(t) & = & \text{exp}\;(e_{⊖\beta }\left(t,t_{0}\right)[g_{0}+\alpha \int_{t_{0}}^{t}e_{⊖\beta }\left(t_{0},\tau \right)\Delta \tau ])\\ & = & \text{exp}\;({\left(\beta +1\right)}^{-(t-t_{0})}[g_{0}+\alpha \sum_{\tau =t_{0}}^{t-1}{\left(1+\beta \right)}^{\tau -t_{0}}])\\ & = & \text{exp}\;({\left(\beta +1\right)}^{-(t-t_{0})}[g_{0}+\alpha \frac{{\left(1+\beta \right)}^{t-t_{0}}-1}{\beta }])\\ & = & \text{exp}\;((g_{0}-\frac{\alpha }{\beta }){\left(\beta +1\right)}^{-(t-t_{0})}+\frac{\alpha }{\beta }), \end{array} \end{array}$$ (52)

respectively for $t\in {\left[t_{0},\infty \right)}_{Z}$ and here we use (9).

In (51) by taking *t*_0_ = 0, *β* = *b* − 1 and *α* = *a* Equation 3.1 is obtained in [11]. Both continuous Gompertz growth curves (18) and (50) are obtained from the IVPs (13) and (43)–(46). If we let *B* = 0, $e\omega_{0}=e^{\frac{\alpha }{\beta }},$ *K* = *β* in (18), and $g_{0}-\frac{\alpha }{\beta }=-1$ in (50), we observe that

$$\begin{array}{c} e.e^{g_{0}}e^{-\frac{\alpha }{\beta }}=e.e^{-1}=1 \end{array}$$

which implies $\omega_{0}=e^{g_{0}}.$ Similarly, the continuous Gompertz curves (23) and (49) are the solutions of the IVPs (21) and (42)–(46). If we let *B* = 0, $\frac{1}{e}\omega_{0}=e^{-\frac{\alpha }{\beta }},$ *β* = −*K* in (23), and $g_{0}+\frac{\alpha }{\beta }=1$ in (49), we observe that

$$\begin{array}{c} e^{-1}.e^{g_{0}}e^{\frac{\alpha }{\beta }}=e^{-1}e=1 \end{array}$$

which implies $\omega_{0}=e^{g_{0}}.$ From these observations, we conclude the 3-parameter first type continuous Gompertz curve and the second type continuous Gompertz curve are identical. However, since such an intimate relation among the discrete curves cannot be observed, considering first and second type discrete Gompertz curves contributes to the literature.

## Logistic dynamic equations

In this section, we derive 4-parameter and 3-parameter Logistic continuous and discrete curves from Logistic dynamic equations.

### 3-Parameter logistic dynamic curves

Since there are two versions of linear equations

$$\begin{array}{c} u^{\Delta }=p\left(t\right)u+f\left(t\right),\;u^{\Delta }=-p\left(t\right)u^{\sigma }+f\left(t\right), \end{array}$$

there are two Logistic dynamic equations

$$\begin{array}{c} L^{\Delta }=\left[⊖\left(p\left(t\right)+f\left(t\right)L\right)\right]L, \end{array}$$ (53)

and

$$\begin{array}{c} L^{\Delta }=\left[p\left(t\right)⊖\left(f\left(t\right)L\right)\right]L, \end{array}$$ (54)

respectively, see [16]. By using the definition of circle minus (6), Logistic dynamic Eqs (53) and (54) turn out to be the typical Logistic differential Eq (3) under certain conditions on *p* and *f* when T=R. In [16], it is shown that the solutions of (53) and (54) subject to

$$\begin{array}{c} L\left(t_{0}\right)=l_{0}\neq 0 \end{array}$$ (55)

are given by

$$\begin{array}{c} L\left(t\right)=\frac{e_{⊖p}\left(t,t_{0}\right)}{\frac{1}{l_{0}}+\int_{t_{0}}^{t}e_{⊖p}\left(\sigma \left(\tau \right),t_{0}\right)f\left(\tau \right)\Delta \tau }, \end{array}$$ (56)

and

$$\begin{array}{c} L\left(t\right)=\frac{e_{p}\left(t,t_{0}\right)}{\frac{1}{l_{0}}+\int_{t_{0}}^{t}e_{p}\left(\tau ,t_{0}\right)f\left(\tau \right)\Delta \tau }, \end{array}$$ (57)

see Theorem 4.2 in [16]. Here, we assume that *p* is regressive, *f* is a rd-continuous function. We now calculate continuous and discrete Logistic solutions in order to compare their data fitting. In population dynamics, one often assumes that there exists a constant *N* ≠ 0 such that *p*(*t*) = *Nf*(*t*) for all t∈T.

**Example 0.16**. Let T=R, *t*_0_ = 1, *f* = *α* and *p* = *β*, where *α* and *β* are constants. Then (56) and (57) turn out to be

$$\begin{array}{c} L=\frac{1}{-\frac{\alpha }{\beta }+(\frac{1}{l_{0}}+\frac{\alpha }{\beta })e^{\beta (t-1)}} \end{array}$$ (58)

and

$$\begin{array}{c} L=\frac{1}{\frac{\alpha }{\beta }+(\frac{1}{l_{0}}-\frac{\alpha }{\beta })e^{-\beta (t-1)}}, \end{array}$$ (59)

respectively.

**Example 0.17**. Let T=Z, *t*_0_ = 1, *f* = *α* and *p* = *β*, where *α* and *β* are constants. (56) and (57) turn out to be

$$\begin{array}{c} L=\frac{1}{-\frac{\alpha }{\beta }+(\frac{1}{l_{0}}+\frac{\alpha }{\beta }){\left(1+\beta \right)}^{t-1}} \end{array}$$ (60)

and

$$\begin{array}{c} L=\frac{1}{\frac{\alpha }{\beta }+(\frac{1}{l_{0}}-\frac{\alpha }{\beta }){\left(1+\beta \right)}^{-t+1}}, \end{array}$$ (61)

respectively.

### 4-Parameter logistic dynamic curves

The 4-parameter Logistic curve

$$\begin{array}{c} \omega \left(t\right)=f-\frac{1}{\frac{b}{f}+(\frac{1}{f-s}-\frac{b}{f})e^{kt}},\;t\in R \end{array}$$ (62)

is introduced and discussed in [15], where *k*, *f*, and *s* are positive constants and *b* is a real number. If we let *b* = 1 in (62), then (62) and (3) are equivalent. Equivalently, we have

$$\begin{array}{c} \omega \left(t\right)=f-\frac{f}{b+(\frac{f}{f-s}-b)e^{kt}},\;t\in R. \end{array}$$ (63)

Motivated by (63), we purpose the 4-parameter logistic dynamic curve as

$$\begin{array}{c} \omega \left(t\right)=f-\frac{f}{b+(\frac{f}{f-s}-b)e_{k}\left(t,0\right)},\;t\in T. \end{array}$$ (64)

To obtain the 4-parameter logistic dynamic equation, we differentiate Eq (64) and derive

$$\begin{array}{ccc} \omega^{\Delta } & = & \frac{f(\frac{f-s}{b}-b)ke_{k}\left(t,0\right)+kfb-kfb}{\left(b+(\frac{f}{f-s}-b)e_{k}\left(t,0\right))(b+(\frac{f}{f-s}-b)e_{k}^{\sigma }\left(t,0\right)\right)}\\ & = & \frac{kf[b+(\frac{f-s}{b}-b)e_{k}\left(t,0\right)]-kfb}{\left(b+(\frac{f}{f-s}-b)e_{k}\left(t,0\right))(b+(\frac{f}{f-s}-b)e_{k}^{\sigma }\left(t,0\right)\right)}\\ & = & \frac{kf}{\left(b+(\frac{f}{f-s}-b)e_{k}^{\sigma }\left(t,0\right)\right)}-\frac{kfb}{\left(b+(\frac{f}{f-s}-b)e_{k}\left(t,0\right))(b+(\frac{f}{f-s}-b)e_{k}^{\sigma }\left(t,0\right)\right)}\\ & = & -k\omega^{\sigma }+kf-\frac{kfb}{\left(b+(\frac{f}{f-s}-b)e_{k}\left(t,0\right))(b+(\frac{f}{f-s}-b)e_{k}^{\sigma }\left(t,0\right)\right)}\\ & + & \frac{kfb}{b+(\frac{f}{f-s}-b)e_{k}\left(t,0\right)}-\frac{kfb}{b+(\frac{f}{f-s}-b)e_{k}\left(t,0\right)}\\ & = & -k\omega^{\sigma }+kf+\frac{kfb}{b+(\frac{f}{f-s}-b)e_{k}\left(t,0\right)}\omega^{\sigma }-\frac{kfb}{b+(\frac{f}{f-s}-b)e_{k}\left(t,0\right)}\\ & = & -k[1-\frac{b}{b+(\frac{f}{f-s}-b)e_{k}\left(t,0\right)}]\omega^{\sigma }+kf[1-\frac{b}{b+(\frac{f}{f-s}-b)e_{k}\left(t,0\right)}]. \end{array}$$ (65)

Hence, we obtain the 4-parameter logistic dynamic equation as:

$$\begin{array}{c} \omega^{\Delta }\left(t\right)=-p\left(t\right)\omega^{\sigma }\left(t\right)+fp\left(t\right),\;t\in T \end{array}$$ (66)

where

$$\begin{array}{c} p\left(t\right)=k[1-\frac{b}{b+(\frac{f}{f-s}-b)e_{k}\left(t,0\right)}],\;t\in T. \end{array}$$ (67)

**Theorem 0.18**. *Let k*, *f*, *s be positive constants*. *Consider the 4-parameter logistic dynamic* Eq (66) *with the initial condition*

$$\begin{array}{c} \omega (0)=s. \end{array}$$ (68)

*Then*,

$$\begin{array}{c} \omega \left(t\right)=f+\left(s-f\right)e_{⊖p}\left(t,0\right),\;t\in T \end{array}$$ (69)

*is the unique solution of the IVP* (66)–(68) *where p is defined as in* (67).

*Proof*. To get the desired result, we use Theorem 0.3. Therefore, we have

$$\begin{array}{ccc} \omega (t) & = & se_{⊖p}\left(t,0\right)+\int_{0}^{t}fpe_{⊖p}\left(t,\tau \right)\Delta \tau \\ & = & se_{⊖p}\left(t,0\right)+\int_{0}^{t}fp\left(\tau \right)e_{p}\left(\tau ,t\right)\Delta \tau \\ & = & se_{⊖p}\left(t,0\right)+f(1-e_{⊖p}\left(t,0\right))\\ & = & f+\left(s-f\right)e_{⊖p}\left(t,0\right),\;t\in T, \end{array}$$

which completes the proof.

**Example 0.19**. If T=Z, then the solution in (69) turns out to be

$$\begin{array}{c} w\left(t\right)=f+\left(s-f\right)\frac{1}{\prod_{\tau =0}^{t-1}[1+k(-\frac{b}{b+(\frac{f}{f-s}-b){\left(1+k\right)}^{\tau }}+1)]}, \end{array}$$ (70)

where we use (9).

Let *b* = 1 in (67). Note that $p=\frac{k}{f}\omega$ and this means that Eq (66) turns out to be

$$\begin{array}{ccc} \omega^{\Delta } & = & -\frac{k}{f}\omega \omega^{\sigma }+k\omega \\ & = & k\omega (1-\frac{\omega^{\sigma }}{f}). \end{array}$$ (71)

If T=R, then we obtain (3) from (71). One of the logistic dynamic equations is (54). By taking (54) into account and using the definition of minus circle, we get

$$\begin{array}{c} L^{\Delta }=(\frac{p-f_{1}L}{1+\mu f_{1}L})x. \end{array}$$

This implies that

$$\begin{array}{c} L^{\Delta }+\mu L^{\Delta }f_{1}L=\left(p-f_{1}L\right)L. \end{array}$$

By the simple useful formula, we have

$$\begin{array}{c} L^{\Delta }+\left(L^{\sigma }-L\right)f_{1}L=\left(p-f_{1}L\right)L. \end{array}$$

Solving the above equation for *L*^Δ^, we get

$$\begin{array}{ccc} L^{\Delta } & = & (p-f_{1}L^{\sigma })L\\ & = & pL(1-\frac{f_{1}}{p}L^{\sigma }). \end{array}$$ (72)

If we take *L* = *ω*, *p* = *k* and $\frac{f_{1}}{p}=\frac{1}{f}$ in (72), then we obtain Eq (71).

At this point the following question arises: Is it possible to find an alternative 4-parameter logistic dynamic equation which turns out to be the equivalent form of the 3-parameter logistic dynamic equation? To find the answer of this question consider Eq (65) where *e*_*k*_ is replaced by $e_{k}^{\sigma }$ in the last two terms of Eq (65). Then we obtain that

$$\begin{array}{ccc} \omega^{\Delta } & = & -k\left(\omega +\mu \omega^{\Delta }\right)+kf-\frac{kfb}{\left(b+(\frac{f}{f-s}-b)e_{k}\left(t,0\right))(b+(\frac{f}{f-s}-b)e_{k}^{\sigma }\left(t,0\right)\right)}\\ & + & \frac{kfb}{b+(\frac{f}{f-s}-b)e_{k}^{\sigma }\left(t,0\right)}-\frac{kfb}{b+(\frac{f}{f-s}-b)e_{k}^{\sigma }\left(t,0\right)}. \end{array}$$ (73)

Solving the above equation for *ω*^Δ^ yields

$$\begin{array}{c} \omega^{\Delta }\left(1+k\mu \right)=-k\omega +kf+\frac{kb}{b+(\frac{f}{f-s}-b)e_{k}^{\sigma }\left(t,0\right)}\omega -\frac{kfb}{b+(\frac{f}{f-s}-b)e_{k}^{\sigma }\left(t,0\right)}, \end{array}$$

or

$$\begin{array}{ccc} \omega^{\Delta } & = & -k\omega [\frac{1-\frac{b}{b+(\frac{f}{f-s}-b)e_{k}^{\sigma }\left(t,0\right)}}{1+k\mu }]+\frac{kf-\frac{kfb}{b+(\frac{f}{f-s}-b)e_{k}^{\sigma }\left(t,0\right)}}{1+k\mu }\\ & = & \left(⊖k\right)\omega [1-\frac{b}{b+(\frac{f}{f-s}-b)e_{k}^{\sigma }\left(t,0\right)}]-f\left(⊖k\right)[1-\frac{b}{b+(\frac{f}{f-s}-b)e_{k}^{\sigma }\left(t,0\right)}]. \end{array}$$

Hence, we get the following logistic dynamic equation

$$\begin{array}{c} \omega^{\Delta }=\left(⊖k\right)q\omega -f\left(⊖k\right)q,\;t\in T, \end{array}$$ (74)

where

$$\begin{array}{c} q\left(t\right)=1-\frac{b}{b+(\frac{f}{f-s}-b)e_{k}^{\sigma }\left(t,0\right)},\;t\in T. \end{array}$$ (75)

**Theorem 0.20**. *Let k*, *f*, *s be positive constants and q be taken as in* (75). *Then*, Eq (74) *with the initial condition* (68) *has the solution*

$$\begin{array}{c} \omega =\left(s-f\right)e_{(⊖k)q}\left(t,0\right)+f,\;t\in T. \end{array}$$ (76)

*Proof*. By using the definition of minus circle (6), we have

$$\begin{array}{ccc} 1+\mu [⊖((⊖k)q)] & = & 1+\mu [⊖\frac{-kq}{1+\mu k}]\\ & = & 1+\mu \frac{kq}{1+\mu k-\mu kq}\\ & = & \frac{1+\mu k}{1+\mu k-\mu kq}. \end{array}$$ (77)

By using Theorem 0.2 and the properties of exponential functions given Theorem 0.1, we arrive at the unique solution as follows:

$$\begin{array}{ccc} \omega (t) & = & se_{(⊖k)q}\left(t,0\right)-f\int_{0}^{t}e_{(⊖k)q}\left(t,\sigma \left(\tau \right)\right)\left(⊖k\right)q\left(\tau \right)\Delta \tau \\ & = & se_{(⊖k)q}\left(t,0\right)-fe_{(⊖k)q}\left(t,0\right)\int_{0}^{t}e_{⊖((⊖k)q)}\left(\sigma \left(\tau \right),0\right)\left(⊖k\right)q\left(\tau \right)\Delta \tau \\ & = & se_{(⊖k)q}\left(t,0\right)\\ & & -fe_{(⊖k)q}\left(t,0\right)\int_{0}^{t}e_{⊖((⊖k)q)}\left(\tau ,0\right)[1+\mu (\tau )⊖((⊖k)q(\tau ))]\frac{-kq(\tau )}{1+\mu (\tau )k}\Delta \tau \\ & = & se_{(⊖k)q}\left(t,0\right)\\ & & +fe_{(⊖k)q}\left(t,0\right)\int_{0}^{t}e_{⊖((⊖k)q)}\left(\tau ,0\right)\frac{1+\mu (\tau )k}{1+\mu (\tau )k-\mu (\tau )kq(\tau )}\frac{kq(\tau )}{1+\mu (\tau )k}\Delta \tau \\ & = & se_{(⊖k)q}\left(t,0\right)+fe_{(⊖k)q}\left(t,0\right)\int_{0}^{t}e_{⊖((⊖k)q)}\left(\tau ,0\right)⊖\left(\left(⊖k\right)q\left(\tau \right)\right)\Delta \tau \\ & = & se_{(⊖k)q}\left(t,0\right)+f(1-e_{(⊖k)q}\left(t,0\right))\\ & = & \left(s-f\right)e_{(⊖k)q}\left(t,0\right)+f,\;t\in T, \end{array}$$

which completes the proof.

**Example 0.21**. If T=Z, then the solution in (76) turns out to be

$$\begin{array}{c} w\left(t\right)=f+\left(s-f\right)\prod_{\tau =0}^{t-1}[1+\frac{k}{k+1}(\frac{b}{b+(\frac{f}{f-s}-b){\left(1+k\right)}^{\tau +1}}-1)]. \end{array}$$ (78)

If we take *b* = 1 in (75), then we get $q=\frac{\omega^{\sigma }}{f}.$ Then Eq (74) turns out to be

$$\begin{array}{c} \omega^{\Delta }=\frac{⊖k}{f}\omega^{\sigma }\left(\omega -f\right). \end{array}$$ (79)

Furthermore, if T=R, (79) turns out to be the logistic differential Eq (3). Note that logistic dynamic Eq (79) is equal neither (53) nor (54). Therefore, (78) with *b* = 1 is different than (60) and (61). It means that obtaining 3-parameter logistic curves from 4-parameter logistic curves yields new 3-parameter logistic discrete curves. The following example consists of two new 3-parameter logistic discrete curves.

**Example 0.22**. If we let T=Z and *b* = 1 in (70) and (78), then we obtain

$$\begin{array}{c} w\left(t\right)=f+\left(s-f\right)\frac{1}{\prod_{\tau =0}^{t-1}[1+k(-\frac{1}{1+(\frac{f}{f-s}-1){\left(1+k\right)}^{\tau }}+1)]}, \end{array}$$ (80)

and

$$\begin{array}{c} w\left(t\right)=f+\left(s-f\right)\prod_{\tau =0}^{t-1}[1+\frac{k}{k+1}(\frac{1}{1+(\frac{f}{f-s}-1){\left(1+k\right)}^{\tau +1}}-1)], \end{array}$$ (81)

respectively.

## Goodness-of-fit test for gompertz and logistic curves and conclusion

The main aim of this study for the statistical analysis of Gompertz and Logistic curves is to determine whether their equations are able to model Pseudomonas Putita and tumor data sets given in [10] and [11]. In order to achieve our goal, *p*-values of the parameters, adjusted R-squared values and six types of errors, namely, RMSE (root mean square error), RRMSE (Relative Root Mean Square Error), MAPE (Mean Absolute Percent Error), MAE (mean absolute error), U1 (Theil inequality coefficient, Theil’s U1), U2 (Theil inequality coefficient, Theil’s U2) for each data set calculated, where

$$\begin{array}{ccc} RMSE=\sqrt{\frac{\sum_{t=1}^{T}e_{t}^{2}}{T}}, & RRMSE=\sqrt{\frac{\sum_{t=1}^{T}{\left|\frac{e_{t}}{y_{t}}\right|}^{2}}{T}}, & MAE=\frac{\sum_{t=1}^{T}|e_{t}|}{T} \end{array}$$

$$\begin{array}{ccc} MAPE=\frac{\sum_{t=1}^{T}\frac{\left|e_{t}\right|}{y_{t}}}{T}, & U1=\frac{\sqrt{\frac{\sum_{t=1}^{T}e_{t}^{2}}{T}}}{\sqrt{\frac{\sum_{t=1}^{T}y_{t}^{2}}{T}}+\sqrt{\frac{\sum_{t=1}^{T}{\hat{y}}_{t}^{2}}{T}}}, & U2=\frac{\sqrt{\frac{\sum_{t=1}^{T}e_{t}^{2}}{T}}}{\sqrt{\frac{\sum_{t=1}^{T}y_{t}^{2}}{T}}} \end{array}$$

Here, *e*_*t*_ shows the error component, *y*_*t*_ the original time series values, ${\hat{y}}_{t}$ the estimated values of the time series, and *T* the number of observations of the series. The criteria to determine an equation showing better performance in terms of goodness of fit is to have statistically significant coefficients; in other words, meaningful *p*-values for each coefficient, the larger adjusted R-squared value and smaller errors (see S1 and S2 Figs). The coefficient estimates of these models are obtained by the Mathematica 11.0 and the Wolfram Language uses “ConjugateGradient”, “Gradient”, “LevenbergMarquardt”, “Newton”, “NMinimize”, and “QuasiNewton” methods.

The curves which successfully model Pseudomonas putita data are 4-parameter first type continuous Gompertz curve (18), 3-parameter first type Gompertz curves (26), (27), (29), Zwietering modification of continuous Gompertz curve (31), Gompertz-Liard curves (35), (38), and 2-parameter second type Gompertz curves 49*α*0, 50*α*0, 51*α*0 and 52*α*0 that are obtained from (49), (50), (51), (52) with *t*_0_ = 1 and *α* = 0. According to the results in S1 Fig Eq (18) has the best fit among Gompertz curves. Therefore, we observe that the performance of 4-parameter Gompertz curves for bacteria is better than 3 and 2-parameter Gompertz growth curves. In addition to these, Eqs (27), (29) and (38) are the 3- parameter discrete Gompertz growth curves are new, thus, contribute to the literature.

When we concentrate attention on the Logistic type growth curves, from S1 Fig, it is apparent that all of the Logistic type equations are successful in modeling the bacteria data set. In addition, growth curves (70), (78), (80), (81) are the new 4-parameter discrete Logistic curves that are obtained in this study. According the results in S1 Fig, among the Logistic type growth curves, 4-parameter Logistic growth curves are better in modeling when it is compared with 3- parameter ones.

Moreover, we can infer that the 4-parameter continuous Logistic curve (63) and (70), (78) model better than Eq (18). Thus, Pseudomonas Putita data is modeled by Logistic type equations better than Gompertz type equations. In addition, Eq (63) highlighted with orange, (70) and (78) highlighted with green in S1 Fig, have the smallest errors among the other Logistic equations. Eqs (70) and (78) have smaller errors after the eighth decimal when they are compared with Eq (63) highlighted yellow in S1 Fig. Therefore, new discrete growth curves (70) and (78) are the best in modeling bacteria data.

Eq (29) highlighted with yellow, (35) highlighted with green, 49*α*0, 50*α*0, 51*α*0 and 52*α*0 highlighted with orange in S2 Fig are the curves that successfully model the tumor data among the Gompertz curves. Eq (35) has the best fit in modeling based on S2 Fig. This equation is the Gompertz Liard continuous equation that was developed for tumor modeling in the literature, so our result is compatible with the one in [14]. On the other hand, 3-parameter discrete Gompertz growth curve (29) also models the tumor data set and this equation is developed in this study as well. In addition, 4-parameter continuous and discrete second type Gompertz curves (51) and (52) are also new. 2-parameter version 51a0 of (51) was studied in [8] as Mirror Gompertz curve. Nevertheless, its discrete version 52*α*0 is a new contribution to the literature. At this point, we declare that 3-parameter Gompertz curves are more successful in modeling tumor data than 4-parameter Gompertz curves. Although the errors of the Logistic type curves are smaller than the errors of Gompertz type curves, all of their parameters are not statistically significant. Therefore, the Gompertz-Liard curve (35) is the best curve in modeling when it is compared with all the other curves and so one can say that Gompertz type curves have better fitting than Logistic type curves for tumor data.

As a result, Logistic curves are better in modeling bacteria data whereas tumor data is modeled better by Gompertz curves. Increasing the number of parameters of Logistic curves give favorable results for bacteria data while decreasing the number of the parameters of Gompertz curves for tumor data turns out to be reasonable. S3 Fig gives us the curve fittings of 4-parameter discrete Logistic curve (70) and 3-parameter discrete Gompertz curve (29) for bacteria data set and also shows the importance of the number of parameters.

## Supporting information

S1 Fig Bacteria. Fitted parameters and statistical error analysis for bacteria. *: significant at.10 level, **: significant at.05 level and ***: significant at.01 level.
(PNG)

S2 Fig Tumor. Fitted parameters and statistical error analysis for tumor data. *: significant at.10 level, **: significant at.05 level and ***: significant at.01 level.
(PNG)

S3 Fig Compare. Compare with 4-parameter discrete Logistic curve and 3-parameter discrete Gompertz curve for bacteria data set.
(PDF)

S1 File Bacteria. Data set for bacteria.
(PDF)

S2 File Tumor. Data set for tumor.
(PDF)

## References

[1] Alverez-Arenas Arturo, Belmonte-Beitia J., and Calvo Gabriel Nonlinear waves in a simple model of hig-grade glioma. Applied Mathemaicts and Nonlinear Sciences. Volume 1: Issue 2, p.405–422 (2016).

[2] Atıcı F. M., Atıcı M., Hrushesky W.J.M., Nguyen N. Modeling Tumor Volume with Basic Functions of Fractional Calculus. Progr. Fract. Differ. Appl. 1, No. 4, 229–241 (2015).

[3] Bassukas I. D., Schultze B. M. The recursion formula of the Gompertz function: A simple method for the estimation and comparison of tumor growth curves. Growth Dev. Aging, Vol.52, (1988), pp.113–122. 3253243

[4] Domingues Jose Sergio. Gompertz Model: Resolution and Analysis for Tumors. Journal of Mathematical Modelling and Application 1, No. 7, 70–77 (2012).

[5] Durbin P. W., Jeung N., Williams M. H., and Arnold J. S. Construction of a Growth Curve for Mammary Tumors of the Rat. Cancer Research. Volume 27, p.1341–1347 (1967). 6047867

[6] Rojas Clara, and Belmonte-Beitia J. Optimal control problems for differential equations applied to tumor growth: state of the art. Applied Mathemaicts and Nonlinear Sciences. Volume 3: Issue 2, p.375–402 (2018).

[7] Dudek Krzysztof, Kedzia Wojciech, Kedzia Emilia, and Kedzia Alicja. Mathematical modelling of the growth of human fetus anatomical structures. Anat Sci Int. 10.1007/s12565-016-0353-y PMC5511295 27393150

[8] Skiadas C.H. Comparing the Gompertz-Type Models with a First Passage Time density Model Advances in Data Analysis. Springer/Birkhauser (2010), pp. 203–209.

[9] Kelley W., and Peterson A. The Theory of Differential Equations: Classical and Qualitative. Springer, Second Edition.

[10] Annadurai G., Rajesh Babu S., Srinivasamoorthy V. R. Development of mathematical models (Logistic, Gompertz and Richards models) describing the growth pattern of Pseudomonas putida(NICM 2174). Bioprocess Engineering, Vol.23, (2000), pp.607–612.

[11] Şengül, S. Discrete fractional calculus and its applications to tumor growth. Master thesis, Paper 161. http://digitalcommons.wku.edu/theses/161, 2010.

[12] Espinosa-Urgel M., Salido A., Ramos J. Genetic Analysis of Functions Involved in Adhesion of Pseudomonas putida to Seeds. Journal of Bacteriology. Volume 182, p.2363–2369 (2000). 10.1128/jb.182.9.2363-2369.2000 PMC111295 10762233

[13] Perz J., Craig A. S., Stratton C. W., Bodner S. J., Philipps W. E. Jr., and Schaffner W. Pseudomonas putida Septicemia in a Special Care Nursery Due to Contaminated Flush Solutions Prepared in a Hospital Pharmacy. Journal of Clinical Microbiology, volume 43, p.5316–5318 (2005). 10.1128/JCM.43.10.5316-5318.2005 PMC1248510 16208007

[14] Tjorve K.M.C., Tjorve E. The use of Gompertz models in growth analyses, and new Gompertz-model approach: An addition to the Unified-Richards family. PLOS ONE 10.1371/journal.pone.0178691. (2017). 28582419 PMC5459448

[15] Wan X., Wang M., Wang G., Zhong W. A new 4 parameter generalized logistic equation and its applications to mammalian somatic growth. Acta Theriologica 45(2):145–153,2000.

[16] Akın-Bohner E. and Bohner M. Miscellaneous Dynamic Equations. Methods and Applications of Analysis. Vol 10, No. 1, pp. 011–030, (2003).

[17] Bohner M. and Peterson A. C. Dynamic Equations on Time Scales: An Introduction with Applications. Birkhauser (2001).

[18] Bohner M. and Peterson A. C. Advances in Dynamic Equations on Time Scales. Birkhauser (2003).

[19] Gibson A.M, Bratchell N., Roberts T.A. Predicting microbial growth: growth responses of salmonellae in a laboratory medium as affected by pH, sodium chloride and storage temperature. Int. J. Food Microbiol. 1988; 6:155–78. 10.1016/0168-1605(88)90051-7 3275296

