# Supplementary figures and images for: Parameter identification for gompertz and logistic dynamic equations
Source: PLoS One. 2020 Apr 9;15(4):e0230582. doi: 10.1371/journal.pone.0230582 (PMC7144974; doi:10.1371/journal.pone.0230582)

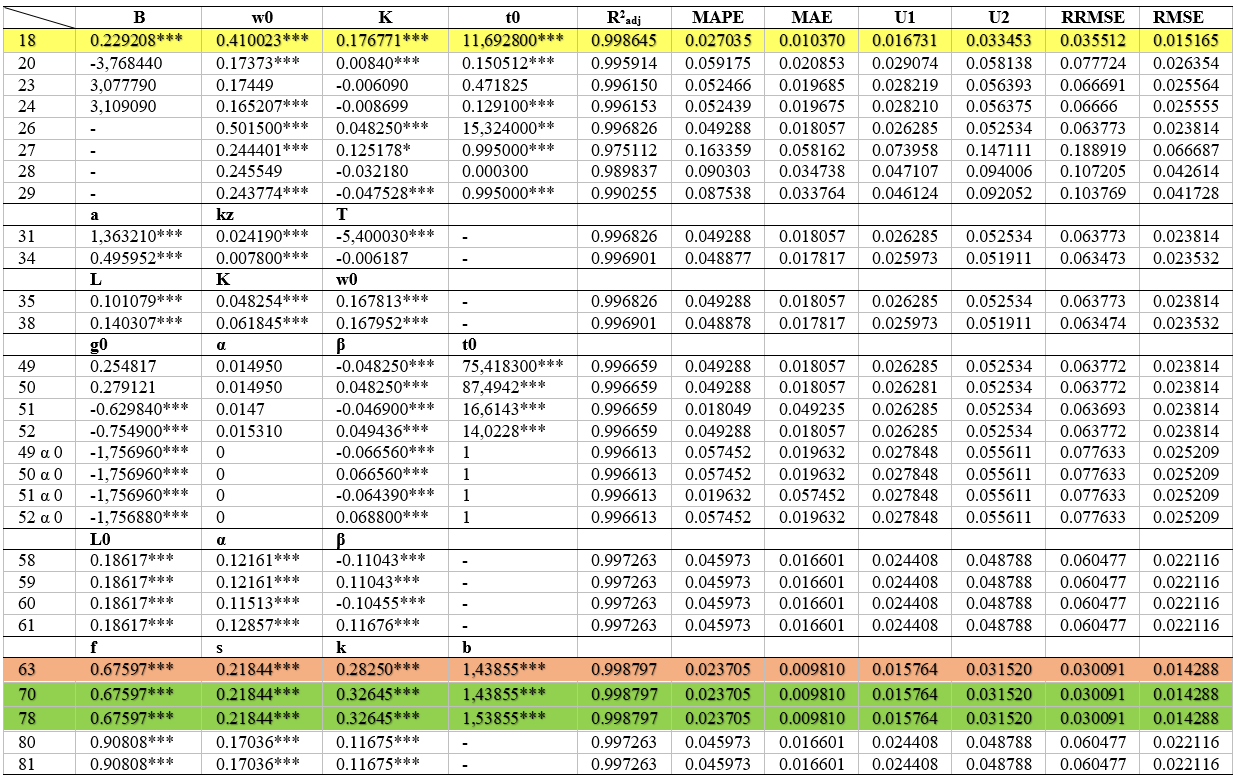

Supplement: S1 Fig — Fitted parameters and statistical error analysis for bacteria. *: significant at.10 level, **: significant at.05 level and ***: significant at.01 level. (PNG) [file pone.0230582.s001.png]

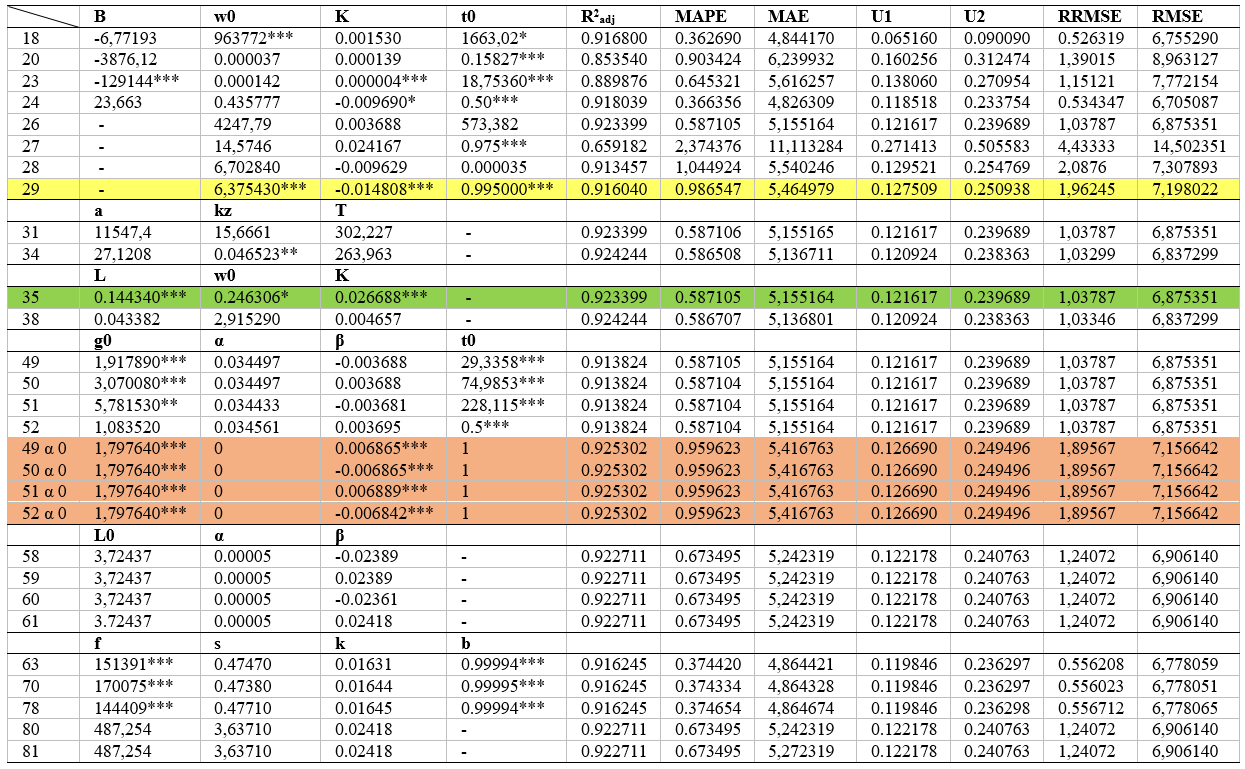

Supplement: S2 Fig — Fitted parameters and statistical error analysis for tumor data. *: significant at.10 level, **: significant at.05 level and ***: significant at.01 level. (PNG) [file pone.0230582.s002.png]

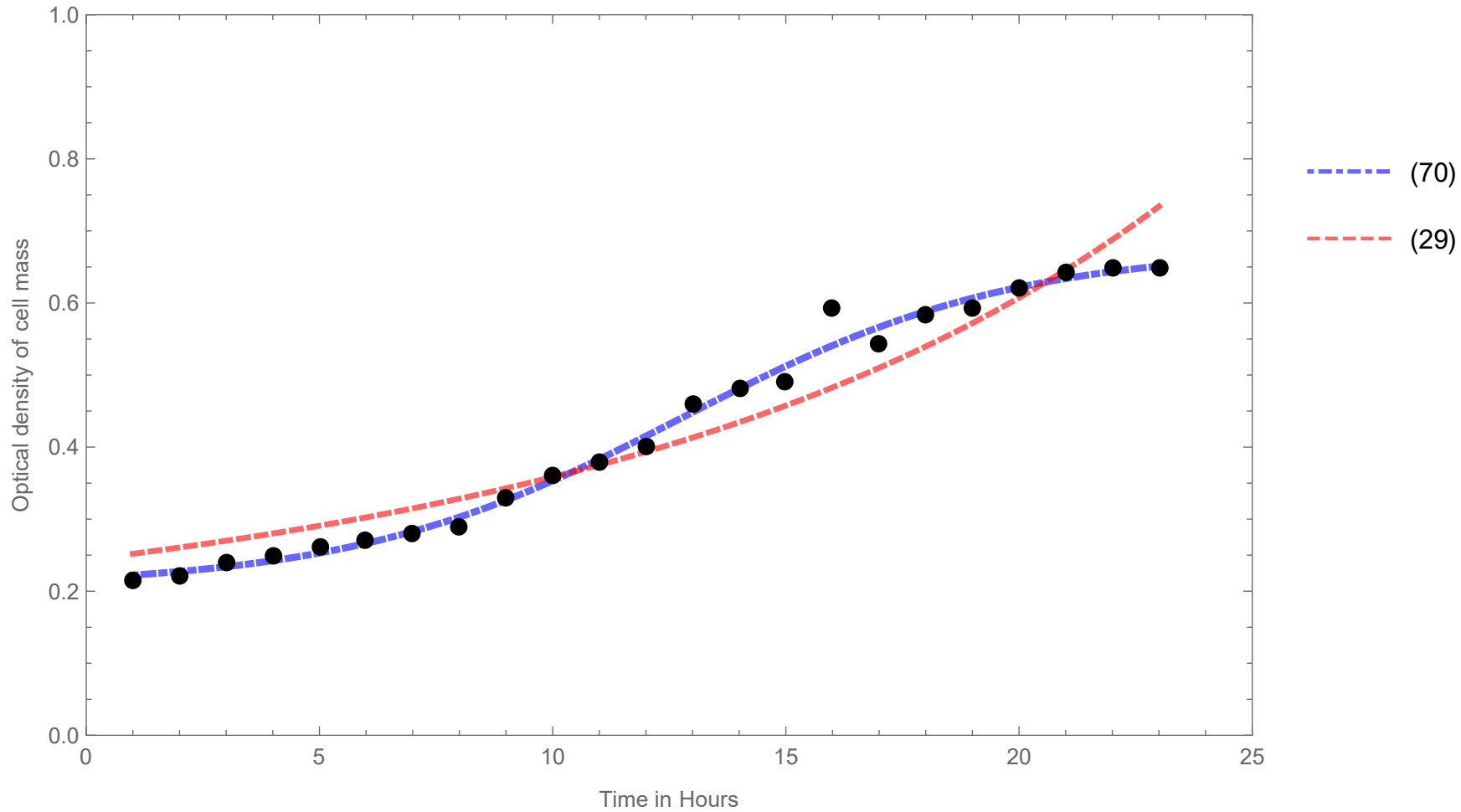

Supplement: S3 Fig — Compare with 4-parameter discrete Logistic curve and 3-parameter discrete Gompertz curve for bacteria data set. (PDF) [file pone.0230582.s003.pdf]
