# Supplementary material for: Parameter identification for gompertz and logistic dynamic equations
Source: PLoS One. 2020 Apr 9;15(4):e0230582. doi: 10.1371/journal.pone.0230582 (PMC7144974; doi:10.1371/journal.pone.0230582)
Supplement: S1 File — Data set for bacteria. (PDF) [file pone.0230582.s004.pdf]

Time (h)	Optical density of cell mass
1	0.216
2	0.220
3	0.240
4	0.250
5	0.260
6	0.270
7	0.280
8	0.290
9	0.330
10	0.360
11	0.380
12	0.400
13	0.460
14	0.482
15	0.492
16	0.592
17	0.544
18	0.584
19	0.594
20	0.620
21	0,644
22	0.648
23	0.648
