# Supplementary material for: Parameter identification for gompertz and logistic dynamic equations
Source: PLoS One. 2020 Apr 9;15(4):e0230582. doi: 10.1371/journal.pone.0230582 (PMC7144974; doi:10.1371/journal.pone.0230582)
Supplement: S2 File — Data set for tumor. (PDF) [file pone.0230582.s005.pdf]

Time (days)	Experimental Data
13	1.19
16	1.4
30.1	5.28
34.3	9.03
51	12.3
51.2	11.34
68.8	31.3
72.5	23.1
92.8	21.2
96	29.8
116.5	65.7
119.6	48.1
